# The Biotic and Abiotic Carbon Monoxide Formation During Aerobic Co-digestion of Dairy Cattle Manure With Green Waste and Sawdust

**DOI:** 10.3389/fbioe.2019.00283

**Published:** 2019-10-29

**Authors:** Sylwia Stegenta-Dąbrowska, Grzegorz Drabczyński, Karolina Sobieraj, Jacek A. Koziel, Andrzej Białowiec

**Affiliations:** ^1^Faculty of Life Sciences and Technology, Wrocław University of Environmental and Life Sciences, Wrocław, Poland; ^2^Department of Agricultural and Biosystems Engineering, Iowa State University, Ames, IA, United States

**Keywords:** CO emissions, aerobic digestion, biomass composting, biowaste, manure, mesophilic conditions, thermophilic conditions

## Abstract

Carbon monoxide (CO), an air pollutant and a toxic gas to humans, can be generated during aerobic digestion of organic waste. CO is produced due to thermochemical processes, and also produced or consumed by cohorts of methanogenic, acetogenic, or sulfate-reducing bacteria. The exact mechanisms of biotic and abiotic formation of CO in aerobic digestion (particularly the effects of process temperature) are still not known. This study aimed to determine the temporal variation in CO concentrations during the aerobic digestion as a function of process temperature and activity of microorganisms. All experiments were conducted in controlled temperature reactors using homogeneous materials. The lab-scale tests with sterilized and non-sterilized mix of green waste, dairy cattle manure, sawdust (1:1:1 mass ratio) were carried out for 1 week at 10, 25, 30, 37, 40, 50, 60, 70°C to elucidate the biotic vs. abiotic effect. Gas concentrations of CO, O_2_, and CO_2_ inside the reactor were measured every 12 h. The CO concentrations observed for up to 30°C did not exceed 100 ppm v/v. For 50 and 60°C, significantly (*p* < 0.05) higher CO concentrations, reaching almost 600 ppm v/v, were observed. The regression analyses showed in both cases (sterile and non-sterile) a statistically significant effect (*p* < 0.05) of temperature on CO concentration, confirming that the increase in temperature causes an increase in CO concentration. The remaining factors (time, O_2_, and CO_2_ content) were not statistically significant (*p* > 0.05). A new polynomial model describing the effect of temperature, O_2_, and CO_2_ concentration on CO production during aerobic digestion of organic waste was formulated. It has been found that the proposed model for sterile variant had a better fit (*R*^2^ = 0.86) compared with non-sterile (*R*^2^ = 0.71). The model predicts CO emissions and could be considered for composting process optimization. The developed model could be further developed and useful for ambient air quality and occupational exposure to CO.

## Introduction

CO is described as the “silent killer”—CO binds to the iron atoms in hemoglobin, with an affinity 200–250 times that of O_2_, and impairs O_2_-carrying capacity of the blood causing hypoxia and highly toxic to living organisms (Kaymak and Basar, 2010; Bürstel et al., 2016). Neurological injuries of CO poisoning are manifested as headaches, dizziness, weakness, nausea, vomiting, disorientation, visual confusion, collapse, and coma. Without immediate treatment, neurological injuries can be fatal (Schallner and Otterbein, 2015). CO is also harmful to the environment, particularly on air quality. Emissions of CO have indirect effects on climate through enhanced levels of tropospheric O_3_ and CH_4_ as a result of its reaction with HO (Hellebrand et al., 2006; Talaiekhozani et al., 2018). It is well-known that CO emissions are associated with anthropogenic sources such as transportation. On the other hand, it is not well-known that biowaste and its treatment (e.g., via composting) are also a source of CO.

Certain conclusions on the mechanisms of CO production in biowaste can be taken from the observation of the biotic and abiotic processes occurring in the soil. CO formation is more likely to occur in dry soils and accelerates with increasing temperature (van Asperen et al., 2015). Research describes the influence of organic matter in the soil, water content, and temperature on CO emissions (Yang et al., 2015; Cowan et al., 2018). Conrad (1996) and Conrad and Seiler (1980) showed that the production of CO is the result of the chemical oxidation of organic matter. It was also shown that higher soil temperature influences the increase in CO production (Conrad and Seller, 1985) and can cause substantial changes to daily emissions (Zepp et al., 1997).

The CO emissions from decaying biomass are rarely raised as a concern on a global scale because of an apparent mitigation mechanism. It has been estimated that microbial uptake of CO in soils is approximately four times greater than emissions. Much of the microbial activity occurs in tropical regions where temperatures are consistently high (>30°C) and damp enough to support thriving microcosm (Liu et al., 2018). CO is also generated during municipal biowaste composting, which was confirmed in our earlier study (Stegenta et al., 2018, 2019a,b), but the biotic and abiotic factors causing it are still not well-understood. Measured CO concentrations were high, thus raising concerns about occupational safety for workers and local air quality. It is generally agreed that CO can be formed in both abiotic and biotic pathways in the aerobic decomposition of organic matter. The abiotic CO formation is attributed to factors such as temperature, UV or visible radiation, organic matter content, humidity or the type of feedstock used (Lee et al., 2012; Fraser et al., 2015; van Asperen et al., 2015). Thermal degradation of carbon compounds can occur at relatively low temperatures (<100°C), resulting in the emission of trace gases such as CO_2_, CH_4_ and also CO. The biotic production of CO by microorganisms is favored under aerobic conditions.

Most of the current knowledge of the biotic/abiotic factors stems from lab-scale experiments. CO concentrations as high as 160 ppmv were measured in lab-scale studies of green waste compost (Hellebrand, 1999; Hellebrand and Kalk, 2001). The highest concentrations were recorded in samples incubated at 35 and 50°C. It was also observed that the concentration of CO was inversely correlated with the CO_2_ concentration in the process air. The maximum CO production occurred when the CO_2_ concentrations were low, while the increase in CO_2_ concentration resulted in reduced CO concentrations (Hellebrand, 1999; Hellebrand and Kalk, 2001). Subsequent experiments suggested that net CO emissions are dependent on temperature, O_2_ availability, and microbial activity. CO concentrations were higher in sterilized waste samples incubated at elevated temperatures and reached up to 1,900 ppmv.

CO can also be consumed in microbiological processes, both under aerobic (Stegenta et al., 2018) and anaerobic conditions (Diender et al., 2015; Oswald et al., 2018). This was also confirmed by studies conducted by Rich and King (1999), who proved that the CO consumption under anaerobic conditions is higher than under aerobic. It was also found that CO is metabolized under all oxidation-reduction conditions. Thus, two types of CO metabolisms can be distinguished: respiratory and fermentative; the first with the participation of exogenous electrons, while the second uses internally generated intermediates as an acceptor of electrons (Diender et al., 2015). An example of respiratory metabolism is the oxidation of CO, coupled with O_2_ reduction (Gullotta et al., 2012). Bacteria of the *Carboxydotrophic* genus, which are aerobic microbes, use CO as a source of C and energy (Pomaranski and Tiquia-Arashiro, 2016). They transfer electrons from CO dehydrogenase (CODH) by catalyzing the oxidation of CO through the respiratory chain, which eventually reduces oxygen. CO_2_ is assimilated as a source of cellular C via the Calvin-Benson-Bassham pathway. These bacteria are especially well-adapted to the role of CO detoxification in the environment because they have a high tendency to absorb CO (Adam et al., 2018). Diender et al. (2015) distinguished and discussed in detail the three main types of fermentative CO metabolism: hydrogenogenesis, methanogenesis, and acetogenesis, generating H_2_, CH_4_, and acetate, respectively.

The process of CO metabolization takes place due to CODH activity in both aerobic and anaerobic conditions. Most methanogenic bacteria contain CODH. However, the enzyme used in aerobic conditions differs from the enzyme under anaerobic conditions in terms of the structure and presence of Ni-Fe clusters in the active sites (Jeong et al., 2015). The function of this enzyme is to mediate between the reduction of CO_2_ to CO, which leads to the formation of the carboxyl group acetyl-CoA. CO is produced from CO_2_ and H_2_ according to following Equation 1 (Esquivel-Elizondo et al., 2017):

(1) $$\begin{array}{r} {\text{CO}}_{2}+{\text{H}}_{2}\to \text{CO}+{\text{H}}_{2}O \end{array}$$

The mechanism of biological production of CO in aerobic conditions is still poorly understood. Rich and King (1999) noted that CO is both consumed and produced by methanogenic, acetogenic, and sulfate-reducing bacteria. However, the precise knowledge of chemical pathways influenced by thermal degradation in natural systems remains elusive due to the numerous interacting processes and conditions observed in composting organic materials, such as microbial activity and the heterogeneous availability of O_2_ (Cowan et al., 2018). However, our previous studies (Stegenta et al., 2018, 2019b) shown that the concentrations of CO in an aerobic composting pile are significant (>1,000 ppm). Additionally, there is an immediate need to drastically reduce emissions in order to limit climate change (Liew et al., 2016). Due to the fact that in Poland many waste treatment plants still facilitate the composting process without environmental controls (i.e., in ambient conditions), knowledge about factors influencing CO production and release can lower the occupational safety risks and protect the air quality. On the other hand, the requirements EU for the protection of the atmosphere (Official Journal of the European Union, 2018) force the process to be hermetic, which with periodic problems with ventilation can be dangerous to the health and even the lives of people working there. As indicated Dey and Dhal (2019), the most important in CO protection is CO monitoring, also in composting process.

This paper aims to explore CO production during aerobic digestion to determine the biotic and abiotic production of CO pathways, and the temporal variation of CO as a function of the process temperature and the presence of O_2_ and CO_2_ in the process gas. Lab-scale studies using co-digestion of dairy cattle manure with grass (green waste) and sawdust using sterile and non-sterile conditions were used. CO production can be biotic (sterile and non-sterile conditions) and abiotic (temperature, humidity, organic matter content); its consumption appears to be strictly a biological process. Additional factors for interpretation are the conditions of the aerobic decomposition process: temperature and availability of O_2_ and CO_2_. Understanding the CO emissions causations, allow the development and introduction of methods to reduce them in treatment composting plants. This, in turn, will improve the protection of both the employees and the air quality. In addition, the improved understanding of fundamental biochemistry of the process could be exploited to maximize the increase of CO formation and its safe capture for production of bio-syngas for energy and industrial purposes.

## Materials and Methods

### Waste Characteristics

The organic waste was a mix of dairy cattle manure, grass clippings, and pine sawdust. These materials represent abundant biowaste resources that are often composted in rural areas or left to decompose. Grass and manure were characterized by high moisture content (Supplementary Table 1). The optimum humidity for the aerobic digestion process is 40–60% (Pikon and Rejman, 2009). Here, the ~59% was obtained by mixing grass, manure with dried sawdust in a mass ratio of 1:1:1 for all experiments. Waste samples were examined before and after the process for moisture content and dry organic matter determined with standard methods (Eaton et al., 1998).

### Experimental Design

The experiment was designed with two independent factors:

- Temperature−10, 25, 30, 37, 40, 50, 60, 70°C (abiotic conditions), and
- microorganism's activity—sterile or non-sterile conditions (biotic conditions).

Data about moisture, organic matter of waste, and O_2_ and CO_2_ concentrations during co-digestion has been collected to evaluate the abiotic/biotic character of the process. The experimental design matrix is shown in Supplementary Table 2.

### Aerobic Digestion

**Figure 1:**
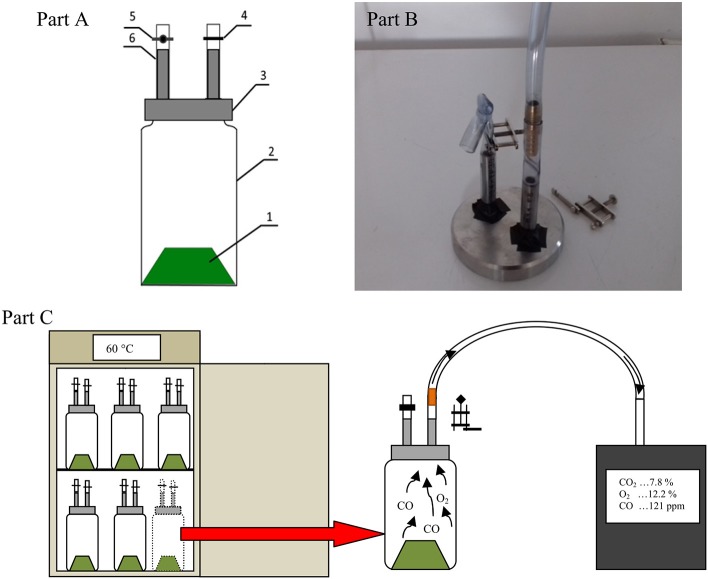
Diagram of the gas production measuring vessel with connectors for gas sampling **(A)**, Photo of connectors with the ignition wire **(B)**, 1, digested material; 2, glass vessel; 3, connectors for gas sampling; 4, hose clamp; 5, Hoffman clamp; 6, silicon tube. Scheme of measuring gases in a reactors **(C)**.

The tests were carried out in controlled temperature 1 L reactors similar to those described by Finstein et al. (1983). All vessels were fitted with a sealed cap with two gas measuring ports equipped with short sections of flex tubing. One port was closed with the Hoffman clamp and a hose clamp. The second port was closed by the Hoffman clamp, which enabled the gas analyzer tube to be connected, for collecting gas samples and carrying out the measurements (Figure 1).

For each trial (*n* = 3), aerobic digestion of 6 samples of mixed organic waste (~50 to 55 g; 3 sterile and 3 non-sterile) was tested at a constant setpoint temperature in the climatic chamber, which also shielded the composting process from light (Supplementary Table 1). Sterile material was prepared by the tyndallization process by placing it three times at 105°C for 1 h for three successive cycles, 24 h apart. The efficiency of tyndallization in samples was checked by measuring oxygen demand (OD) with Oxi-Top (WTW, Germany) sets. A resulting constant O_2_ level indicated full sterilization. Samples of non-sterile material were prepared on the last day of samples sterilization.

The headspace of the reactors was ventilated after each gas measurement to maintain aerobic digestion conditions in the presence of oxygen. The manner of air supply varied for sterile and non-sterile samples. Reactors with non-sterilized waste were opened after measurement for several minutes; they were sealed again and returned for further incubation due to the much higher OD. A tube with a disposable sterile syringe filter was connected to reactors with sterilized waste, similar to gas concentration measurements. After sealing the connection and opening the Hoffman clamp, the vacuum created during the gas measurements pulled the fresh air through the filter into the inside of the reactor. Then, the hose was clamped and the syringe filter was removed. In addition, to avoid contamination of sterile samples, we used a cotton pad soaked with denatured rinsing surface, as suggested by Phillip et al. (2011). Other reactor parts were also treated in the same way before trials (Figure 1).

### Analyses of Process Gas Concentrations

Measurements of selected gases (CO, O_2_, and CO_2_) concentrations during the aerobic digestion process were carried out for a week, twice a day. The measurement was performed in 3 replicates for both treatments−3 for sterile samples and 3 for non-sterilized samples. The first measurement was performed 12 h after the start of incubating at the selected incubation temperature. The gas concentrations were measured using a Kimo KIGAZ 200 (Kimo Instruments, Chevry-Cossigny, France) exhaust gas analyzer (Technical Data Sheet, 2018) for approximately 50 s to stabilize the measured values. The Kigaz 300 analyzer was factory-calibrated by comparing with standards of metrology laboratories (Figure 1C).

### Statistical Analysis

The results were subjected to statistical analysis using the Statistica 12.5 software (Cracow, Poland). The experimental data were subjected to the statistical analyses to understand the variation and correlation among the different parameters from 7 days of the composting process. The principal component analysis (PCA) was used first and the impact of individual factors (temperature, O_2_ & CO_2_ content, and time) on CO concentration was analyzed with the partial least squares (PLS) regression models. The PCA was used to screen for the most important factors influencing CO formation among temperature, O_2_ & CO_2_ content, and time – with different scales). The factors we defined as dimensions in PCA. The use of PCA (which is a linear dimensionality reduction algorithm) facilitated dimensions standardization and reduction of the initial complexity of the linear model. Additionally, the PLS was used to generate the first concept of the linear model and to improve the understanding of the influence of particular factors and for improved visualization of these relationships. The multiple polynomial regression analysis was performed at the α = 0.05 significance level to build the initial mathematical model for biotic and abiotic conditions separately. The polynomial regression analysis was used because composting process has non-linear character.

## Results

### The CO Concentration Changes During Aerobic Digestion

**Figure 2:**
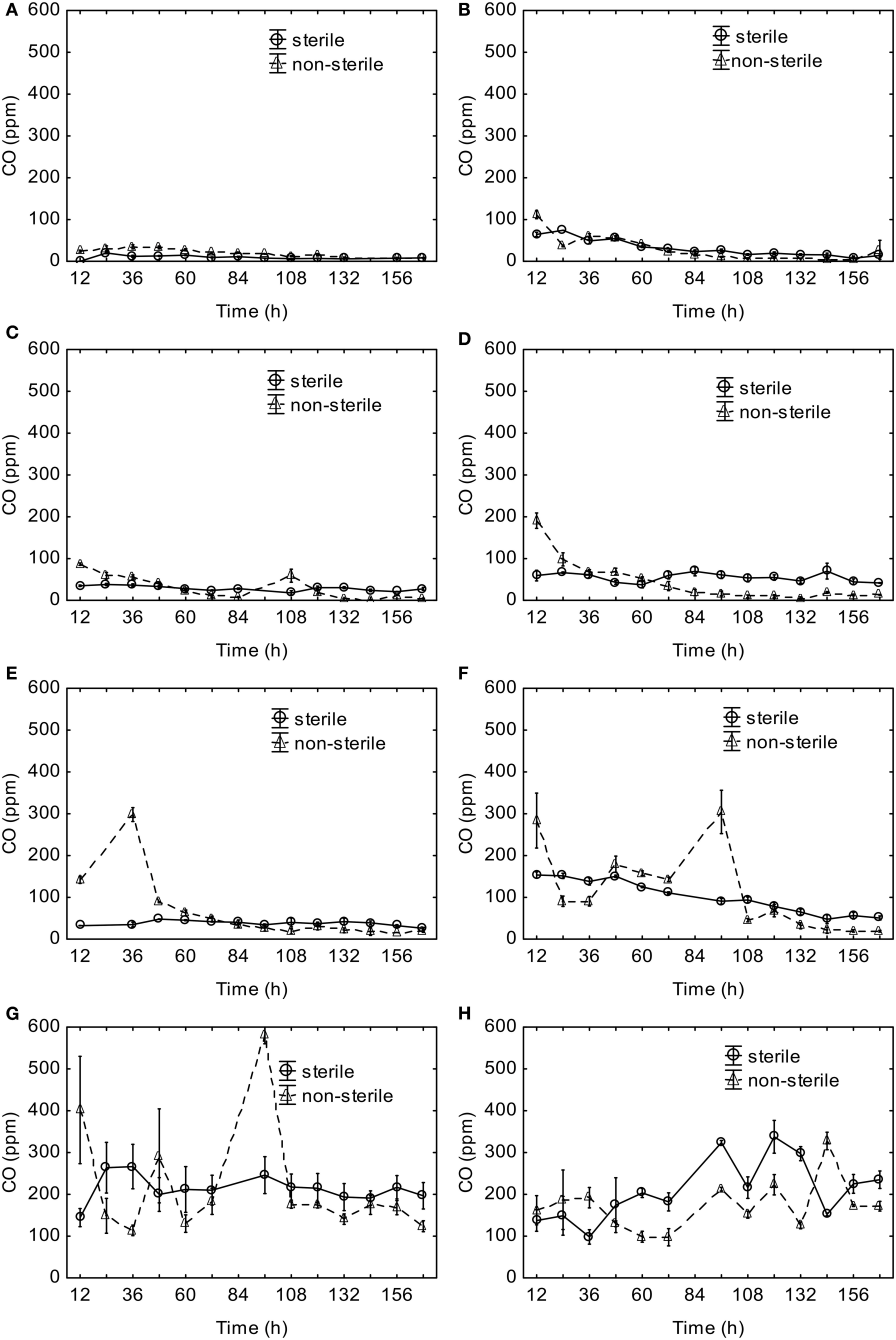
Headspace CO concentrations during aerobic digestion at **(A)** 10°C, **(B)** 20°C, **(C)** 30°C, **(D)** 37°C, **(E)** 40°C, **(F)** 50°C, **(G)** 60°C, **(H)** 70°C, (mean ± standard error).

The observed CO concentrations in the headspace increased with the increasing temperature of aerobic digestion (Figure 2). The CO concentrations observed for incubation temperatures up to 30°C did not exceed 100 ppm v/v (Figures 2A–C). The CO concentrations in the sterile material were stable over time. However, the headspace CO concentrations in the non-sterile material were higher at the beginning, and they slowly declined as the experiment progressed (Figures 2A–E) for all temperatures up to 40°C. For 50 and 60°C, significantly higher CO concentrations were observed at the beginning and the 96^th^ h (Figure 2G). For 70°C, a similar CO trend was observed in both sterile and non-sterile material, with higher values observed in the sterile material (Figure 2H). With the exception to 70°C, higher CO concentrations were generated by the non-sterile material.

### Temporal Variation in O_2_ and CO_2_ Concentrations During Aerobic Digestion

Temporal variations of O_2_ concentrations had noticeable trends with increased temperature and varied depending on the initially sterile and non-sterile conditions. The O_2_ and CO_2_ concentrations in the headspace of the sterile material at 10°C was stable and ~ 20.1% v/v O_2_ and 0.8% v/v CO_2_ (Supplementary Figure 1A). For the non-sterile material, the O_2_ concentration started at 15%, dropped to 11% v/v after 24 h, and then recovered slowly, and ~84 h reached about 16% v/v, where it remained constant until the end.

Similarly, the O_2_ concentration in the sterile material at 20°C, was very stable and ~20.1% v/v (Supplementary Figure 1B). The O_2_ concentration in the reactor with non-sterilized waste was initially remarkably low (~1% v/v), then gradually increased to 12% v/v at 48 h. Then, after 84 h, it slowly increased to ~14% v/v at the end of the experiment. The experiment performed at 30°C had high and stable O_2_ concentrations in the sterile material (Supplementary Figure 1C). The O_2_ concentration in the non-sterile material started at 18% v/v, and it slowly decreased to ~12% v/v at its end. The content of CO_2_ was changing oppositely to O_2_ changes, showing a strong correlation between those two gases content in the process gas in both variants (Supplementary Figures 1B,C).

O_2_ concentrations during the experiment conducted at 37°C showed uncommonly high variability in reactors with both sterile and non-sterile material (Supplementary Figure 1D). The O_2_ concentration in the sterile material decreased from about 17% v/v to about 12% v/v during the experiment. In the non-sterile material, the O_2_ concentration for the first 2 days was at an exceptionally low level of ~3%, and then it increased to ~10% v/v and varied from 4 to 10% v/v until the end of the experiment.

Tests carried out at 40°C had a high O_2_ (~20% v/v) content in the reactors with a sterile material and low in a non-sterile material (2–4% v/v) (Supplementary Figure 1E). At 50°C, the O_2_ content in the sterile material ranged from 19.5% v/v to ~16% at the end of the process (Supplementary Figure 1F). The O_2_ concentration in the non-sterile material was much lower throughout the process (~4.5% v/v). The O_2_ concentration at 60°C in the sterile material was ~18.5% v/v throughout the experiment (Supplementary Figure 1G). In the non-sterile material, the O_2_ concentration varied between 7 to 10% v/v. A completely different pattern was noticed during the experiment conducted at 70°C (Supplementary Figure 1H). The O_2_ concentration was at a similar level (around 19–20% v/v) in both sterile and non-sterile material.

CO_2_ concentrations in the headspace of sterile conditions were typically much lower compared with non-sterile conditions (with the exception of 70°C where they were nearly identical). In both cases, the CO_2_ concentrations were fairly stable during the entire digestion except for 70°C where the changes and variability were unusually high similar to O_2_. The content of CO_2_ fluctuations showed a strong correlation with the O_2_ depletion due to biochemical or thermochemical processes of organic matter oxidation.

### Changes in Waste Properties During Aerobic Digestion

The loss of moisture was observed in most of the post-digestion samples (Supplementary Table 3) compared to the initial values (Supplementary Table 3). The only exceptions were the case of non-sterile material at 10°C and 25°C and sterile material at 10°C (Supplementary Table 3). Also, lower moisture content (by ~3–4%) was observed in the sterile material compared with the non-sterile material (Supplementary Table 3) at 10–40°C. At temperatures above 50°C the trend was reversed, i.e., ~4–5% higher moisture was observed in the sterile material.

Organic matter content decreased for all digestions as all temperatures (Supplementary Table 3) compared to the input material (Supplementary Table 2). Higher removal rates of organic matter were observed for the non-sterile material. No effect of temperature on the degradation of organic substances was observed, probably due to the relatively short digestion time (1 week).

### The Influence of Temperature, CO_2_ and O_2_ on CO Concentration in Process Gas During Aerobic Digestion

#### PCA analysis

**Figure 3:**
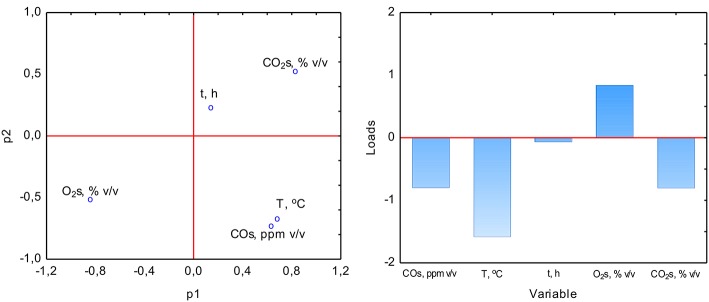
Loads scattering plot p1 vs. p2 **(left)** with a loads value of particular variables in the PCA analysis **(right)** of variables obtained in sterile samples.

**Figure 4:**
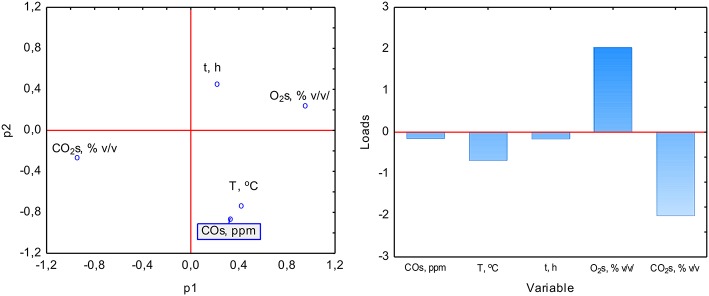
Loads scattering plot p1 vs. p2 **(left)** with a loads value of particular variables in the PCA analysis **(right)** of variables obtained in non-sterile samples.

The PCA analysis was used with the NIPALS algorithm. Results showed that time played a minor role in the overall variable model in the case of sterile samples. However, the temperature had the strongest impact on CO formation and was directly proportional to CO concentration. The strength of the O_2_ and CO_2_ concentration was lower, but these parameters were inversely correlated (Figure 3). In the case of non-sterile samples, the concentration of O_2_ and CO_2_ exerted the most substantial influence on the shape of the interdependencies between variables, while the nature of dependence between variables remained unchanged (Figure 4). The impact of time as a factor has increased under biotic conditions.

#### PLS Analysis

**Figure 5:**
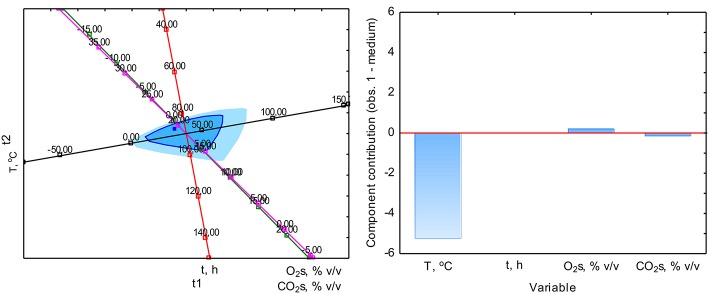
Analysis of the impact of individual factors on CO concentration during composting in sterile samples by the method PLS **(left)** with the load's value of particular variables **(right)**.

**Figure 6:**
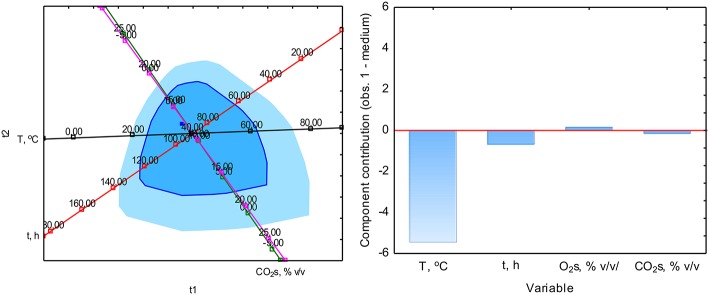
Analysis of the impact of individual factors on CO concentration during composting in non-sterile samples by the method PLS **(left)** with a loads value of particular variables **(right)**.

The study of the influence of particular factors on CO concentration by the PLS method showed an increase in the importance of temperature in CO production during aerobic digestion in the case of sterile samples (Figure 5). In the case of non-sterile samples, a similar increase in the role of temperature was observed, while the duration of the process gained significance, which may be related to the biological nature of CO formation or metabolism and microorganisms growth kinetics as elements affecting the net CO production (Figure 6). The appearance of the influence of time may be a factor showing the presence of biotic determinants on CO formation during composting. Thus, a multiple polynomial regression was investigated to build a more realistic model due to the fact that composting is not a linear process.

#### Multiple Regression Analysis

In the case of sterile material, a statistically significant effect (*p* < 0.05) of temperature on CO concentration was demonstrated. Regression coefficients were positive, which confirms that the increase in temperature (biotic conditions) causes an increase in CO concentration (Table 1). The remaining factors were not statistically significant, and the degree of explanation for the variance of *R*^2^ was 0.864 (Supplementary Table 4). In the case of non-sterile samples, the influence of the duration of the process became evident in addition to the dominant role of temperature in the net CO formation. At the same time, it has been shown that CO concentration decreases over time (Table 1). In the case of non-sterile samples, the explanation of variance *R*^2^ was 0.712 (Supplementary Table 4). The equations for the process carried out under sterile (2) and non-sterile (3) conditions are presented below (according to Table 1):

(2) $$\begin{array}{r} CO\left(ppmv\right)=\left(-1.921\cdot T\right)+{\left(0.066\cdot T\right)}^{2} \end{array}$$

(3) $$\begin{array}{r} CO\left(ppmv\right)=\left(-8.321\cdot T\right)+{\left(0.153\cdot T\right)}^{2}+\left(-0.910\cdot t\right) \end{array}$$

where:

*T* – temperature,°C

*t* – time, h.

**Table 1:** Evaluation of parameters by multiple polynomial regression—parameterization with sigma limitations—concentration of CO in sterile and non-sterile material.

**Type of waste treatment**	**Effect**	**COs, ppmv (Param.)**	**COs, ppmv (Standard deviation)**	**COs, ppmv(t)**	**COs, ppmv (p)**	**-95.00% confidence interval**	**+95.00% confidence interval**	**COs, ppm v/v[Beta (β)]**	**COs, ppm v/v (Standard deviation β)**	**−95.00% confidence interval**	**+95.00% confidence interval**
Sterile	Intercept	884.598	416.518	2.124	0.035	64.575	1704.622				
	T,°C	−1.921	0.708	−2.712	0.007	−3.316	−0.527	−0.410	0.151	−0.708	−0.112
	T,°C^2^	0.066	0.008	8.125	0.000	0.050	0.082	1.185	0.146	0.898	1.473
	t, h	0.237	0.235	1.009	0.314	−0.225	0.699	0.134	0.133	−0.128	0.396
	t, h^2^	−0.002	0.001	−1.443	0.150	−0.004	0.001	−0.192	0.133	−0.453	0.070
	O_2_s, % v/v	−111.268	81.797	−1.360	0.175	−272.307	49.771	−4.083	3.002	−9.993	1.826
	O_2_s, % v/v^2^	3.337	2.979	1.120	0.264	−2.527	9.201	3.574	3.190	−2.706	9.854
	CO_2_s, % v/v	40.432	46.543	0.869	0.386	−51.201	132.064	1.365	1.571	−1.728	4.458
	CO_2_s, % v/v^2^	−4.464	3.419	−1.306	0.193	−11.196	2.268	−1.991	1.525	−4.994	1.012
Non-sterile	Intercept	504.635	132.936	3.796	0.000	243.031	766.240				
	T,°C	−8.321	2.025	−4.110	0.000	−12.306	−4.337	−1.466	0.357	−2.168	−0.764
	T,°C^2^	0.153	0.026	5.940	0.000	0.102	0.204	2.255	0.380	1.508	3.002
	t, h	−0.910	0.387	−2.352	0.019	−1.670	−0.149	−0.430	0.183	−0.789	−0.070
	t, h^2^	0.002	0.002	1.202	0.230	−0.002	0.007	0.216	0.180	−0.138	0.569
	O_2_ns, % v/v	−6.113	15.417	−0.396	0.692	−36.451	24.226	−0.339	0.854	−2.019	1.342
	O_2_ns, % v/v^2^	−0.765	0.659	−1.160	0.247	−2.062	0.533	−0.915	0.789	−2.468	0.637
	CO_2_ns, % v/v	−18.152	15.826	−1.147	0.252	−49.297	12.993	−0.935	0.815	−2.540	0.669
	CO_2_ns, % v/v^2^	0.121	0.765	0.159	0.874	−1.383	1.626	0.126	0.790	−1.429	1.680

## Discussion

### Temporal Changes in the Concentration of O_2_, CO_2_, and CO

The highest CO_2_ concentrations were observed in the headspace of non-sterile material at 40°C. This is in line with the results of Lee et al. (2012), who reported substantial increases in both the CO_2_ and CO production rates with temperatures above 35°C, and contrary to the studies performed by Eklind et al. (2007) and Miller (1992), who noticed the highest emissions at 55°C. This proves that the optimal temperature for the biowaste composting process depends on the content of easily biodegradable ingredients and other substances affecting the process (Eklind et al., 2007). The observed CO_2_ and O_2_ concentrations in both sterile and non-sterile material were similar to those observed by Phillip et al. (2011) in an experiment performed under laboratory conditions with municipal solid waste. Presence of other gases is an important factor affecting CO production. It was observed that the presence of O_2_ increased CO concentrations in sterile samples (Phillip et al., 2011). Research carried out by Haarstad et al. (2006) suggests a correlation between emitted CO and H_2_S. CO concentrations from the decomposition of kitchen waste mixed with green waste ranged from 21 to 194 ppmv. The CO concentration reached its maximum of 2,022 ppmv (average 486 ppmv) when lime was added to the waste. The organic matter load was quite high in this case, which caused high O_2_ consumption and the development of methanogenic bacteria under anaerobic conditions.

Hellebrand and Schade (2008) showed that the highest CO concentrations were observed during the first few days of the process and then gradually reduced. This trend was maintained in the samples regardless of the temperature at which the experiment was conducted. It was noticed that the CO concentration increased again at about 5 days into the process only at lower temperatures (5, 20, and 35°C). However, Kirschbaum (2010) provided additional evidence that the observed gas production was not microbially mediated, as the optimum temperature for typical microbial activity was ~35°C.

Our laboratory experiment proved that the production process of CO depends on biotic conditions (the activity of microorganisms in the material) and abiotic conditions—it is stimulated by the temperature increase. A similar trend, by Hellebrand and Kalk (2001). The maximum CO concentrations for the first few hours of the process explain CO production via the thermochemical route. Then, when the maximum activity of microorganisms is reached, low O_2_ concentrations were observed, which should have a negative effect on CO production due to lack of O_2_ for the process of thermochemical oxidation. Lee et al. (2012) speculated that trace gas release from aerobic waste degradation might be due to thermal excitation of organic functional groups by lowering the barrier to reactivity with reactive oxidized species (ROS) or simple thermal decomposition. On the other hand, CO is an energy source in anaerobic conditions for many bacteria with the genus *Carboxydotrophic* (Pomaranski and Tiquia-Arashiro, 2016). Both of these processes lead to a reduction in CO concentration in the decomposed material.

The concentration of CO in the soil subjected to the sterilization process by means of autoclaving or radiation increased CO production in fresh and rehydrated soil, indicating that the production does not have a biological basis, but rather chemical oxidation (Moxley and Smith, 1998). Because many CO-metabolizing bacteria are thermophilic (growth range from 55 to 82°C), CO concentration should be lower under thermophilic conditions in non-sterile material compared with the sterile conditions. The activity of microorganisms would be demonstrated by increased CO_2_ content and lower CO concentration on process gas (Techtmann et al., 2009). Lower CO concentrations in the non-sterile material under temperatures of 60 and 70°C were also observed in our experiment, indicating that some CO could have been consumed by CO-metabolizing bacteria. However, due to lower production of CO_2_ at 60 and 70°C (indicative of the lower activity of microorganisms) compared to measurements performed at lower temperatures, it is suspected that this production was mostly of thermochemical origin and the consumption by metabolizing bacteria was rather small.

Haarstad et al. (2006) observed that CO concentrations under aerobic conditions ranged from 21 to 194 ppm, with an average of 101 ppm. The elevated concentrations of CO to over 2,000 ppm were noticed by the authors explaining the intensive decomposition of organic matter initiated by the addition of lime. Hellebrand and Kalk (2001) noticed that the CO emission per mass of the substrate was the highest at 35°C - 18.6 mg CO·kg^−1^ substrate. The CO production dropped to 11 mg CO·kg^−1^ at 50°C, with a simultaneous increase in CO_2_ emissions from 110 mg·kg^−1^ at 35°C to 173 mg·kg^−1^ at 50°C, suggesting increased activity of microorganisms metabolizing CO. At 65°C, a decrease in CO and CO_2_ content were observed compared to 50°C, but CO_2_ production was still significant, which did not go beyond the optimal range of CO metabolizing bacteria. Low CO concentrations in low-temperature sterilized samples are consistent with the literature presented earlier (Hellebrand et al., 2006). In this study, we observed the maximum CO concentrations in temperature 60°C, at the lowest observed O_2_ concentrations, and the highest CO_2_ concentrations (Supplementary Figure 1G), which is the result of high activity of microorganisms at this temperature—optimal for composting process. The multiple polynomial regression analysis (Table 1) also shows the dominant influence of abiotic factors (temperature) on the CO production process, regardless of the influence of biotic factors (the use of sterility or not).

The CO concentration could also be affected by waste moisture (~60%), which, as in the study by Moxley and Smith (1998), were beyond the optimal range of the microorganism which can produce CO in aerobic conditions. On the other hand, recent research showed that the relationship between soil moisture and CO production is less consistent (Cowan et al., 2018). Further research is required to investigate specifically the relationship between waste moisture and its influence on CO formation. The content of organic matter and the rate of its decomposition, which increases with temperature, could have a more significant impact on the presented experiment.

During the composting of green waste, it was shown that CO concentrations were 50 ppm at the beginning, similarly to Andersen et al. (2010), which decreased to the 50th day of the process virtually to zero, similar to our experiment. However, it should be emphasized that after 100 d of the process, a repeated increase in CO concentration to about 80 ppm was observed, when the temperature in the heap increased to 80°C. In studies carried out by Hellebrand (1999) at low CO_2_ concentrations, it was shown that CO production at 35°C increased significantly and reached a maximum value exceeding 200 μg CO·h^−1^.

The observed (temporarily) increased CO concentrations from non-sterile samples resulted from insufficient O_2_ in research vessels (Haarstad et al., 2006). Increased CO production for non-sterile material could be the result of the development of methanogenic bacteria, which, as explained by Xavier et al. (2018), can produce CO at hypoxic conditions. Esquivel-Elizondo et al. (2017) showed that the formation of CO by methanogenic bacteria was dependent on the presence of H_2_ and CO_2_. This is in contradiction with the previously quoted literature (e.g., Hellebrand and Kalk, 2001) and our own research where higher O_2_ concentrations resulted in increased CO production. It follows that CO production can take place in both aerobic and anaerobic conditions. However, the mechanisms of CO production remain speculative. Rich and King (1999) suggested that the influence of O_2_ on CO production may result from the acceleration of fatty acid oxidation and the decomposition of free radicals into humic substances—substances necessary for the production of CO by microorganisms under aerobic conditions. However, no literature data were found, that would comprehensively describe the effect of low and high O_2_ concentrations on CO production during aerobic decomposition of waste. Hence further detailed studies on the mechanism and dependence of CO production on the availability of O_2_ are necessary.

### The Impact of Individual Factors on CO Concentration

To date, the impact of process parameters such as O_2_ and CO_2_ concentrations or temperature on CO concentration, have not been described comprehensively. Few attempts to correlate the effect of individual parameters on CO concentration were reported (Haarstad et al., 2006; Andersen et al., 2010; Phillip et al., 2011). Andersen et al. (2010) reported a high linear correlation of CO with CO_2_ (*R*^2^ = 0.99) in the first few minutes of the composting process carried out in laboratory conditions in a non-sterile material. Our study did not show such a strong relationship, likely because of the different time scale (1 week). In our research, the greatest impact on CO concentration was attributed to temperature. Phillip et al. (2011) also confirmed that CO concentration increases with the temperature and had the strongest effect on increasing emissions in the first days of the composting process. On the other hand, a low correlation with CH_4_ (*R*^2^ = 0.02) recorded by Haarstad et al. (2006) may prove that anaerobic conditions in which CH_4_ is the main product, have a low impact on CO production. Navarro et al. (2014) stated that methanogenic bacteria have a CODH enzyme that allows the use of CO as a C source and its oxidation, which may indicate that it can be metabolized under suitable conditions. However, the efficiency of methanogenesis with CO as a substrate is not too high, which explains its effect on CO concentration.

Our research shows that the relationships between target parameters are much more complicated and rarely have a linear character. The PCA, PLS analysis and regression analyses show that under certain conditions, the number of elements influencing CO production can be limited (as in the case of sterile material, where only the temperature is significant). In the case of aerobic digestion, the activity of microorganisms is one of the critical elements of the process. Hence it cannot be omitted from this type of research. In the case of non-sterile samples, the role of time in the formation of CO was demonstrated, which is likely related to the deterioration of organic matter by the 1^st^ order reaction and kinetics of microorganism's growth. In this case, the proposed multiple polynomial models (with provided regression coefficients) should be further developed and optimized. The influence of time on CO formation may be a factor showing that biological processes start to play an important role in CO formation during composting. Research in this area should be continued, especially considering the kinetics of CO formation under various operational conditions.

## Summary

It was shown that CO production had both abiotic and biotic character and observed CO concentrations depended mainly on the temperature and changed within the time during the organic waste aerobic decomposition process. It was shown that under sterile conditions the influence of temperature on CO production was more significant than in samples not subjected to sterilization, in which the influence of process duration was visible, which indicates the role of microorganisms in net CO production. It has been shown that when biological processes are involved in organic matter decomposition and CO formation the influence of factor of time increases. This research is the preliminary determination of a multiple polynomial regression model describing the effect of temperature, O_2_ and CO_2_ concentration, and time on CO production during aerobic digestion of organic waste. Determined model parameters should be further researched and optimized. Future research should consider the role of CO consumption as a factor that can effectively reduce CO emissions from the composting process.

## Data Availability Statement

All datasets generated for this study are included in the article/Supplemental Material.

## Author Contributions

AB, SS-D, GD, and KS: conceptualization. AB, SS-D, and GD: methodology. AB and JK: formal analysis. AB, GD, and JK: validation. SS-D, GD, and KS: investigation. KS and SS-D: resources. AB and KS: data curation. KS and SS-D: writing–original draft preparation. AB, GD, and JK: writing–review and editing. SS-D and KS: visualization. AB and JK: supervision.

### Conflict of Interest

The authors declare that the research was conducted in the absence of any commercial or financial relationships that could be construed as a potential conflict of interest.
