# Supplementary material for: The Biotic and Abiotic Carbon Monoxide Formation During Aerobic Co-digestion of Dairy Cattle Manure With Green Waste and Sawdust
Source: Front Bioeng Biotechnol. 2019 Oct 29;7:283. doi: 10.3389/fbioe.2019.00283 (PMC6828980; doi:10.3389/fbioe.2019.00283)
Supplement: Supplementary file 1 [file Data_Sheet_1.docx]

Supplementary Material

**Supplementary Figure 1**. Headspace O_2_ and CO_2_ concentrations in aerobic digestion (legend on part h indicates sterile conditions: ○ - O_2_, ● - CO_2_, and non-sterile conditions: Δ- O_2_, ▲- CO_2_) and A 10 °C, B 20 °C, C 30 °C, D 37 °C, E 40 °C, F 50 °C, G 60 °C, H 70 °C, (mean ± standard error).

**Supplementary Table** **1**. Characteristics of individual substrates and their mixtures

| Substrate | Moisture, % | Organic matter, % d.m. |
| --- | --- | --- |
| Grass | 79.53±2.6 | 93.35±0.3 |
| Dairy cattle manure | 83.82±1.6 | 74.80±0.9 |
| Sawdust | 0.00±0.0 | 75.56±0.4 |
| Mixture of all substrates | 59.0±4.1 | 90.2±0.7 |

**Supplementary Table** **2**. Experimental design matrix

| Type of waste | Process temperature,  ^o^C | Waste processing before the process |
| --- | --- | --- |
| manure + grass + sawdust | 10, 25, 30, 37, 40, 50, 60, 70 | sterile, non-sterile |

**Supplementary Table** **3**. Material properties after aerobic digestion (mean ± standard deviation)

| Proces Temperature,  °C | Moisture (%) | | Organic matter (% d.m.) | |
| --- | --- | --- | --- | --- |
|  | sterile | non-sterile | sterile | non-sterile |
| 10 | 60.25±1.75 | 63.02±3.40 | 89.28±0.67 | 90.23±0.72 |
| 25 | 57.92±1.38 | 61.29±0.73 | 90.26±2.30 | 89.48±2.64 |
| 30 | 52.83±2.33 | 57.45±1.57 | 91.47±0.63 | 89.18±0.32 |
| 37 | 56.66±4.09 | 58.10±2.28 | 92.29±0.24 | 89.99±0.51 |
| 40 | 54.50±1.85 | 59.85±2.63 | 90.84±1.80 | 87.89±0.56 |
| 50 | 50.42±0.60 | 45.99±1.64 | 89.88±0.30 | 89.45±0.46 |
| 60 | 44.06±1.69 | 49.69±0.80 | 89.19±0.32 | 89.63±0.37 |
| 70 | 50.09±0.35 | 53.94±1.16 | 91.01±0.47 | 89.57±0.22 |

**Supplementary Table** **4**. Sum of squares (SS) test for a full model relative to the concentration of CO in sterile and non-sterile material

| Type of waste treatment | dependent variable | multiple R | multiple R^2^ | corrected  R^2^ | SS  Model | df  Model | MS  Model | SS  Residue | df  Residue | MS  Residue | F | p |
| --- | --- | --- | --- | --- | --- | --- | --- | --- | --- | --- | --- | --- |
| Sterile (s) | COs, ppm v/v | 0.864 | 0.747 | 0.740 | 1586380 | 8 | 198298 | 537040 | 271 | 1982 | 100.1 | 0.00 |
| non-sterile (ns) | COns, ppm v/v | 0.712 | 0.507 | 0.494 | 1677813 | 8 | 209727 | 1633762 | 300 | 5446 | 38.5 | 0.00 |
